# 

**Published:** 1897-04

**Authors:** 


### BOOKS RECEIVED.

The Diseases of the Stomach. By Dr. C. A. Ewald, Extraordinary Professor of Medicine at the University of Berlin ; Director of the Augusta Hospital, etc. Translated and edited, with numerous additions, from the third German edition, by Morris Manges, A. M., M. D., Assistant Visiting Physician to Mount Sinai Hospital ; Lecturer on General medicine at the New York Polyclinic, etc. Second revised edition. New York : D. Appleton & Co. 1897.

Lectures on Appendicitis and Notes on Other Subjects. By Robert T. Morris, A. M., M. D., Fellow of the New York Academy of Medicine, American Association of Obstetricians and Gynecologists, American Medical Association ; Member of the New York State and County Medical Societies, Society of Alumni of Bellevue Hospital, Linnean Society of Natural History, etc. Second edition, revised and enlarged. With illustrations by Henry Macdonald, M. D. New York : G. P. Putnam's Sons, 27 West Twenty-third street. London : The Knickerbocker Press, 24 Bedford street, Strand. 1897.

How to Live Longer and Why We Do Not Live Longer. By J. R. Hayes, M. D., Medical Examiner Bureau of Pensions, Department of the Interior, Washington, D. C. ; Late Surgeon United States Volunteers ; Late Medical Director Department of the Potomac, G. A. R. Philadelphia : J. B. Lippincott Co. 1897.

Lectures on Renal and Urinary Diseases. By Robert Saundby, M. D., Edin., F. R. C. P., Lond. ; Emeritus Senior President of the Royal Medical Society ; Fellow of the Royal Medico-Chirurgical Society ; Professor of Medicine in Mason College, Birmingham, etc., etc. With numerous illustrations. Second edition. Small 8vo, pp. xii.—434. Philadelphia : W. B. Saunders. 1897.

A Practical Treatise on Diseases of the Skin. For the use of students and practitioners. By J. Nevins Hyde, A. M., M. D., Professor of Dermatology and Venereal Diseases in Rush Medical College,

Chicago, and Frank H. Montgomery, M. D., Lecturer on Dermatology and Venereal Diseases, Rush Medical College, Chicago. New (fourth) edition. In one octavo volume of 815 pages, with 110 engravings and twelve full-page plates, four of which are colored. Cloth, $5.25 ; leather, $6.25. Philadelphia and New York : Lea Brothers & Co., Publishers. 1897.

Seventeenth Annual Report of the State Board of Health of New York. With fourteen maps of sewer systems and sewage disposal works (in separate volume). Transmitted to the legislature February 17, 1896. Albany : Wynkoop Hallenbeck Crawford Company, State Printers. 1896.

The Year-Book of Treatment for 1897. A critical review for practitioners of medicine and surgery. Crown octavo, 488 pages. Cloth, $1.50. Philadelphia and New York : Lea Brothers & Co. 1897.

Inebriety : Its Source, Prevention and Cure. By Charles Follen Palmer. Sexto-decimo, pp. 109. Price, 50 cents. New York : Fleming H. Revell Company, 112 Fifth avenue.

The Blind as Seen Through Blind Eyes. By Maurice de la Sizeranne. Authorised translation from the second French edition, by F. Park Lewis, M. D., Member of the Board of Trustees of the New York Institution for the Blind. 12mo, pp. 170. New York : G. P. Putnam's Sons, 27 West Twenty-third street. 1897.

The Retrospect of Practical Medicine and Surgery. Being a half-yearly journal containing a retrospective view of every discovery and practical improvement in the medical sciences. Edited by James Braithwaite, M. D., London, Obstetric Physician and Surgeon to the Leeds General Infirmary, etc., etc. Assisted by E. F. Trevelyan, M. D., London, Assistant Physician to the Leeds General Infirmary. Volume CXIV·, January, 1897. New York : G. P. Putnam's Sons, 27 West Twenty-third street.

Hypnotism and its Application to Practical Medicine. By Otto Georg Wetterstrand, M. D., Member of the Society of Swedish Physicians at Stockholm, etc., etc. Authorised translation, by Henrik G. Petersen, M. D. Together with medical letters on hypno-suggestion, etc. Octavo, pp. 183. New York : G. P. Putnam's Sons, 27 West Twenty-third street. 1897.

Transactions of the Eighteenth Annual Meeting of the American Laryngological Association, held in the City of Pittsburg, Pa., May 14, 15 and 16, 1896. Henry L. Swain, M. D., Secretary. New York : D. Appleton & Co. 1897.

Diseases of the Eye and Ophthalmoscopy. A handbook for physicians and students. By A. Eugen Fick, M. D., University of Zurich. Authorised translation by Albert B. Hale, A. B., M. D., one of the Ophthalmic Surgeons to the United Hebrew Charities ; Consulting Ophthalmic Surgeon to Charity Hospital, Chicago. Octavo, pp. xvi.—488. With a glossary and 158 illustrations. Price, $4.50. Philadelphia : P. Blakiston, Son & Co., 1012 Walnut street. 1896.

---

THE *Albany Medical Annals* appeared in the beginning of the year with a new garb and a general improved appearance. Its last edition is a special double number for March and April, 1897, in which it publishes in full a discussion on the relation of impure water to disease, which took place at the annual meeting of the Medical Society of the State of New York, January 26–28, 1897, and which was organised by Dr. Henry Hun, of Albany.

